# Modulation of Bcl-x Alternative Splicing Induces Apoptosis of Human Hepatic Stellate Cells

**DOI:** 10.1155/2016/7478650

**Published:** 2016-08-07

**Authors:** Lin Wu, Chengqiong Mao, Xin Ming

**Affiliations:** Division of Molecular Pharmaceutics, UNC Eshelman School of Pharmacy, University of North Carolina, Chapel Hill, NC 27599, USA

## Abstract

Liver fibrosis is a major cause of morbidity and mortality worldwide due to chronic viral hepatitis and, more recently, from fatty liver diseases. Activation and proliferation of hepatic stellate cells (HSCs) represent a key aspect of fibrogenesis and are associated with progressive reduction of HSC apoptosis. Bcl-x, an antiapoptotic member of Bcl-2 gene family, plays a role in apoptosis regulation in mammalian cells. Through alternative splicing, the Bcl-x gene yields two major protein isoforms with opposing functions, antiapoptotic Bcl-x_L_ and proapoptotic Bcl-x_S_. This study aimed to investigate the role of Bcl-x and its alternate splicing in HSC apoptosis. The results indicated that the expression of Bcl-x_L_ was dramatically higher than Bcl-2 in activated human HSCs. The relative expression of Bcl-x_L_ over Bcl-x_S_ increased gradually when HSCs were activated in cell culture, which was consistent with the increase in apoptosis resistance of activated HSCs. Redirection of Bcl-x splicing by an antisense oligonucleotide from the antiapoptotic isoform to the proapoptotic isoform induced death of HSCs without other apoptosis stimuli. We conclude that Bcl-x plays a role in regulation of HSC apoptosis and modulation of Bcl-x alternative splicing may become a novel molecular therapy for liver fibrosis.

## 1. Introduction

Liver fibrosis represents a pathological response to chronic liver damage and is associated with inflammation and excessive accumulation of extracellular matrix (ECM) [1, 2]. There is mounting demand for effective therapies for liver fibrosis and its end-stage sequela of cirrhosis as they have become a major source of morbidity and mortality in humans [3]. According to the WHO Global Burden of Disease study for the years 2000–2012, 1,021,000 people are estimated to have died from cirrhosis worldwide in 2012, accounting for 1.8% of total deaths and a 14.5% increase from 2000. An additional 740,000 deaths occur due to liver cancer, which usually arises from cirrhosis. The prevalence of liver fibrosis is ever increasing in a population faced with precipitously rising obesity rates and thereby increasing rates of important risk factors such as nonalcoholic fatty liver disease and nonalcoholic steatohepatitis [4, 5]. Approved therapies for the treatment of fibrosis are currently nonexistent, which produces a serious economic burden for healthcare systems worldwide and generates enormous demands for new medical therapies to halt or reverse fibrosis [4, 6, 7].

The excessive accumulation of ECM in liver is a hallmark of liver fibrosis and hepatic stellate cells (HSCs) are the major producers of the fibrotic ECM [2]. HSCs are activated in response to chronic liver injury and develop a myofibroblast-like phenotype associated with increased proliferation and collagen synthesis [4]. As activated HSCs are the major producers of the fibrotic ECM and the most downstream cellular effectors of liver fibrosis, survival of HSCs can determine the progress of liver fibrosis and thereby becomes another hallmark of liver fibrosis [8, 9]. For example, fibrosis progression was associated with progressive reduction of HSC apoptosis in patients with chronic HCV [10]. On the other hand, fibrosis resolution has been demonstrated through a mechanism of HSC apoptosis in an animal model [11]. Thus, stimulating apoptosis of HSCs is considered as a possible effective strategy to achieve liver fibrosis resolution [6, 9, 12, 13]. Activated human HSCs are resistant to many proapoptotic stimuli, such as serum deprivation, Fas-ligand, and toxic levels of bile acids, and this may result from increased expression of antiapoptotic protein Bcl-2 [14].

**Figure 1:**
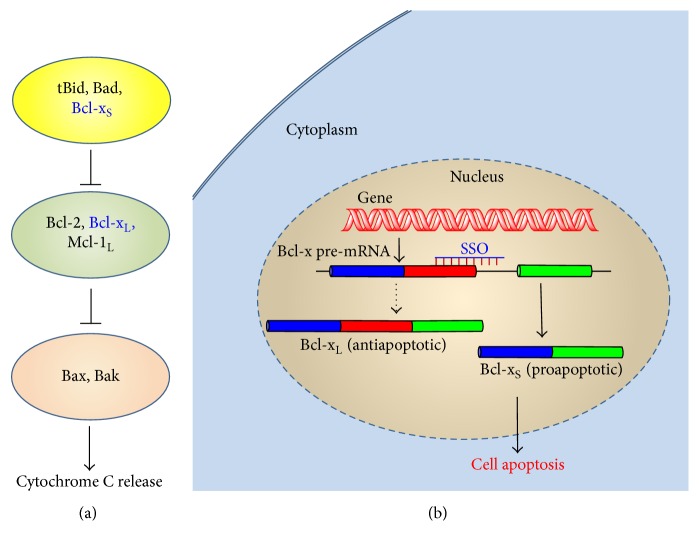
Regulation of apoptosis by Bcl-2 family proteins. (a) The apoptotic pathway is initiated when apoptotic stimuli activate BH3-only members and indirectly induce apoptosis through antagonism of antiapoptotic Bcl-2 proteins. (b) Direction of Bcl-x alternative splicing by SSO. Alternative splicing of Bcl-x pre-mRNA yields antiapoptotic Bcl-x_L_ and proapoptotic Bcl-x_S_. SSO redirects the splicing machinery and results in a simultaneous decrease in production of Bcl-x_L_ and increase in production of Bcl-x_S_.

The “apoptotic trigger” in mammalian cells is controlled by counterbalancing members of the Bcl-2 family, including the proapoptotic Bax-like proteins, the antiapoptotic Bcl-2 homologs, and the proapoptotic BH3-only proteins (Figure 1(a)) [15]. The archetypal member Bcl-2 along with its closest relatives including Bcl-x_L_ and Mcl-1_L_ is inhibitors of apoptosis, acting in large part by binding to and thereby suppressing two proapoptotic triggering proteins (Bax and Bak); the latter are embedded in the outer mitochondrial membrane (OMM). When the proapoptotic BH3-only proteins including tBid and Bim relieve them from inhibition by their antiapoptotic Bcl-2 homologs, Bax and Bak disrupt the integrity of the OMM, causing the release of proapoptotic signaling proteins such as cytochrome c and thereafter a cascade of downstream cellular changes associated with the apoptotic program [15]. The balance of BH3-only and antiapoptotic Bcl-2 proteins determines whether the cell will commit to apoptosis. The significance of specific Bcl-2 family members in apoptosis resistance depends on their relative expression levels and is specific to cell type. Alternative splicing of some Bcl-2 family members is a key mechanism for regulation of these proteins [16]. Through alternative splicing, the Bcl-x gene yields two major protein isoforms with opposing functions, antiapoptotic Bcl-x_L_ and proapoptotic Bcl-x_S_ [17, 18]. Modulation of the alternative splicing of Bcl-x can change ability of mammalian cells to resist apoptosis (Figure 1(b)) [16, 19]. Splice-switching oligonucleotide (SSO) is a type of antisense oligonucleotides that can modulate alternative splicing by hybridizing to pre-mRNA sequences and blocking access of various splicing factors [20]. Thus, a SSO can redirect Bcl-x splicing from antiapoptotic Bcl-x_L_ to proapoptotic Bcl-x_S_ and thereby induce apoptosis of cancer cells [21]. By eliminating an overexpressed prosurvival splice variant and inducing an apoptotic splice variant simultaneously, Bcl-x SSO can potentially achieve greater pharmacological effect than a Bcl-x_L_ inhibitor that can only act on a single function [20, 22]. Redirection of alternative splicing using antisense oligonucleotides has achieved initial clinical success in treating the patients with Duchenne muscular dystrophy in a phase II clinical trial [23].

The purpose of this study was to investigate the role of Bcl-x and its alternative splicing in apoptosis of HSCs. Further, we also aimed to test the feasibility of stimulating HSC apoptosis using a Bcl-x SSO.

## 2. Methods

### 2.1. Materials

The SSOs were 2′-O-Me oligonucleotide with phosphorothioate linkages and were custom synthesized by Integrated DNA Technologies, Inc. (Coralville, IA, USA). The Bcl-x SSO (5′-TGGTTCTTACCCAGCCGCCG-3′) was targeted to the 5′-splice site of exon II in Bcl-x pre-mRNA [21, 24] SSO654 (5′-GCTATTACCTTAACCCAG-3′), which was targeted to intron two of aberrantly spliced *β*-globin and has no effect on Bcl-x pre-mRNA splicing, serving as negative control. Total RNA of freshly isolated human HSCs and hepatocytes were obtained from ScienCell Research Laboratories (San Diego, CA, USA).

### 2.2. Cell Culture

Human primary HSCs were purchased from ScienCell Research Laboratories (San Diego, CA, USA) and were grown at 37°C, 5% CO_2_ in recommended Stellate Cell Medium provided by ScienCell according to the methods reported previously [14, 25, 26]. Consistent with these previous studies using human primary HSCs, the cells showed phenotypic features of fully activated HSCs between passages 3 and 7 and thus the cells at passage 7 were used in the experiments of SSO transfection.

### 2.3. Real-Time PCR

Total RNA was extracted using an RNA purification Kit (Thermo Scientific), and cDNA was synthesized from total RNA using an Enhanced Avian RT First Strand Synthesis Kit (Sigma-Aldrich, St. Louis, MO, USA). Real-time PCR reactions were performed on an ABI Prism 7900 system (Applied Biosystems). Predesigned TaqMan primer and probe sets were used for quantification of Bcl-x_L_ and Bcl-x_S_ (Life Technologies Product IDs Hs00236329_m1 and Hs00169141_m1, resp.). The probes span the boundary of exons II and III of Bcl-x_L_ and Bcl-x_S_, respectively. Because exon II in Bcl-x_L_ has extra 138 bp fragment compared to that of Bcl-x_S_ [27], the primers and probes only detect the corresponding splice variants of Bcl-x gene. TaqMan human *β*-actin endogenous control (Applied Biosystems) was used for quantification of *β*-actin in each sample and served as an internal control. The target gene expression levels of samples were calculated with the Comparative C_T_ Method (ΔΔC_T_ Method) and were expressed as fold changes over controls.

### 2.4. Bcl-x Splicing by SSO

Transfection of human HSC was performed using the Amaxa nucleofection technology according to a method reported previously [28]. Briefly, 1 million of HSCs were nucleofected in T solution of the Amaxa cell optimization kit plus SSOs (0.125–1 nmol) using the U-25 program. The transfected cells were immediately transferred into 6-well plates containing prewarmed culture medium. One day after transfection, total RNA was isolated using TRIZOL reagent (Life Technologies, USA). Relative mRNA expression levels of Bcl-x_L_ and Bcl-x_S_ were analyzed by gel electrophoresis. For gel electrophoresis, the PCR products (Bcl-x_L_ 351 bp and Bcl-x_S_ 162 bp) were separated on 1.7% gels and bands were visualized and quantified on a Molecular Imager® Gel Doc*™* XR System (Bio-Rad, Hercules, CA). The ratio of Bcl-x_S_/Bcl-x_L_ was determined by dividing the intensity of the 162 bp band by the intensity of the 351 bp band.

### 2.5. Evaluation of Cytotoxicity and Apoptosis

The cytotoxicity of the SSOs was evaluated using Alamar Blue assay. In brief, human HSCs transfected with Mock SSO and Bcl-x specific SSO were seeded into 96-well plates at the density of 5,000 cells/well and the cells were further incubated for 72 h. Alamar Blue reagent was added and incubated for 2 h. The plate was read in FLUOstar Omega microplate reader (BMG LabTech, Cary, NC, USA) set at 540 nm excitation wavelength and 590 nm emission wavelength.

To evaluate HSC apoptosis, human HSCs were seeded into 24-well plates at a density of 50,000 cells/well after transfection with the SSOs using Amaxa. After 24 h, 48 h, and 72 h, cells were harvested and a mitochondrial membrane potential/annexin V apoptosis kit (Life Technologies, USA) was used to identify apoptotic cells according to the manufacturer's manual. Apoptosis was measured by flow cytometry using a LSRII cell analyzer (Becton-Dickenson, San Jose, CA, USA).

For confocal fluorescence microscopy observation, the cells were seeded in Lab-Tek 8 Chambered cover glasses (Nalge Nunc) at a density of 50,000 cells/well after Amaxa transfection. After 48 h, cell nuclei were labeled by incubation with 10 *μ*M of DRAQ5*™* (BioStatus, UK) for 20 min at room temperature. Cells were then imaged by a laser scanning confocal microscopy (Olympus FV1000 MPE). Doses of Mock SSO and Bcl-x specific SSO used in the apoptosis assays were 0.5 nmol.

### 2.6. Data Analysis

Data are expressed as mean ± SD from three measurements unless otherwise noted. Statistical significance was evaluated using *t*-test for two-sample comparison or ANOVA followed by Dunnett's test for multiple comparisons. The data were analyzed with GraphPad Prism 5 (GraphPad Software, Inc., La Jolla, CA, USA).

## 3. Results

### 3.1. Increased mRNA Expression Ratio of Bcl-x_L_ and Bcl-x_S_ in Activated Human HSCs

**Figure 2:**
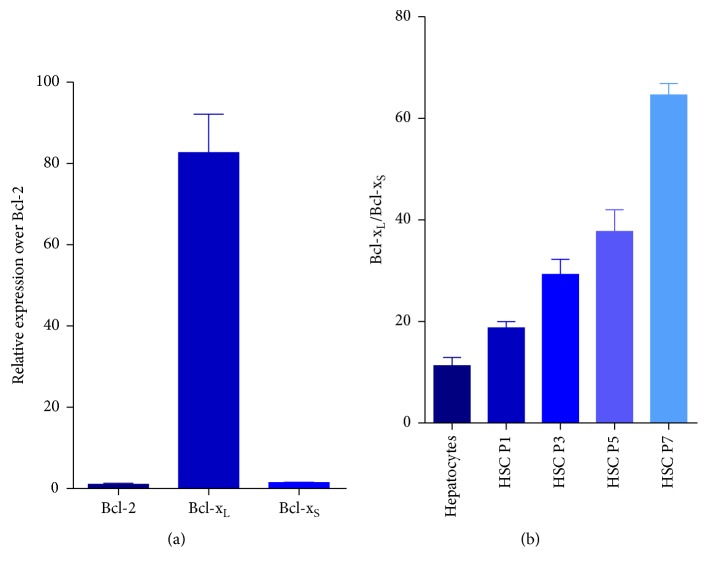
Gene expression of Bcl-x_S_ and Bcl-x_L_ in human HSCs. Real-time PCR analysis of RNA extracted from human HSCs of different passages. (a) The gene expression of Bcl-2, Bcl-x_L_, and Bcl-x_S_ was compared in activated human HSCs (passage 7). (b) The relative expression ratio of Bcl-x_L_ and Bcl-x_S_ was compared in human hepatocytes and human HSCs at different passages.

Total RNAs of HSCs in different passages were collected and subjected to real-time PCR analysis. As shown in Figure 2(a), the gene expression of Bcl-x_L_ was 83-fold higher than Bcl-2 in activated human HSCs (passage 7). The results also showed that the relative expression ratio of Bcl-x_L_ and Bcl-x_S_ increased in later passages, from 18 at passage 1 to 65 at passage 7 (Figure 2(b)). Further, the ratio of Bcl-x_L_ and Bcl-x_S_ of freshly isolate human hepatocytes was significantly lower than that of activated HSCs (passage 7) (Figure 2(b)).

### 3.2. Bcl-x Splicing Shift in SSO Transfected Human HSCs

**Figure 3:**
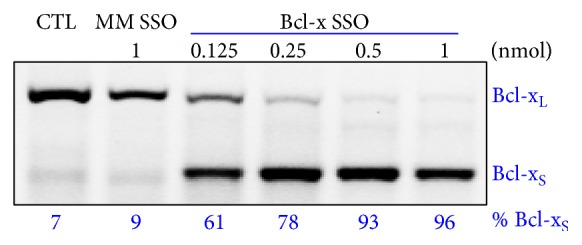
SSO induced Bcl-x splice-switching in human HSCs. RT-PCR analysis of Bcl-x mRNA from cells transfected with control and Bcl-x SSO was performed 24 h after transfection. The Bcl-x SSO dose-dependently induced switching of Bcl-x mRNA splicing from antiapoptotic Bcl-x_L_ to proapoptotic Bcl-x_S_.

Human HSCs were transfected with increasing amounts of Bcl-x SSO, which targeted the downstream alternative 5′-splice site in Bcl-x pre-mRNA. This SSO could block the targeted splice site and induce a shift in the splicing pathway from Bcl-x_L_ to Bcl-x_S_ mRNA [21]. Total cell RNA 24 h after treatment was subjected to RT-PCR analysis. It is shown in Figure 3 that Bcl-x SSO treatment led to a dose-dependent shift in splicing of Bcl-x pre-mRNA from Bcl-x_L_ to Bcl-x_S_. At the highest dose of 1 nmol, there was over a 96% shift from Bcl-x_L_ to Bcl-x_S_ isoform. By contrast, there was no shift in Bcl-x pre-mRNA splicing in the cells treated with the negative control SSO654 even at the highest dose.

### 3.3. Cytotoxicity Induced by SSO Treatment

**Figure 4:**
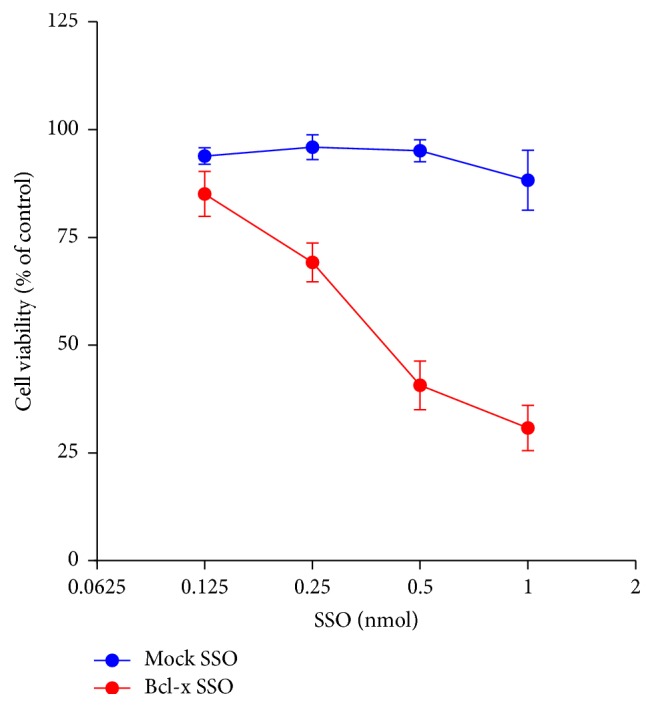
Bcl-x SSO dose-dependently induced cytotoxicity in HSCs. Human HSCs were transfected with Mock SSO or Bcl-x SSO using an Amaxa machine. Then cells were seeded into 96-well plates at the density of 5000 cells/well. After incubation of 72 hours, Alamar Blue reagent was added and incubated for another 2 hours. The plate was then read for fluorescence value using a microplate reader. Bcl-x SSO showed a dose-dependent cytotoxicity. Data are expressed as mean ± SD (*n* = 3).

From Figure 3, we confirmed that shifting splicing of Bcl-x pre-mRNA led to simultaneous downregulation of antiapoptotic Bcl-x_L_ and upregulation of proapoptotic Bcl-x_S_. To evaluate the effect of Bcl-x SSO on cell survival, an Alamar Blue assay was performed 72 h after Amaxa transfection of SSOs. As shown in Figure 4, the Bcl-x specific SSO dose-dependently induced cytotoxicity in human HSCs. Using the same transfection method, the control SSO654 did not cause cytotoxicity even at high dose.

### 3.4. Apoptosis of Human HSCs Caused by Bcl-x SSO Transfection

**Figure 5:**
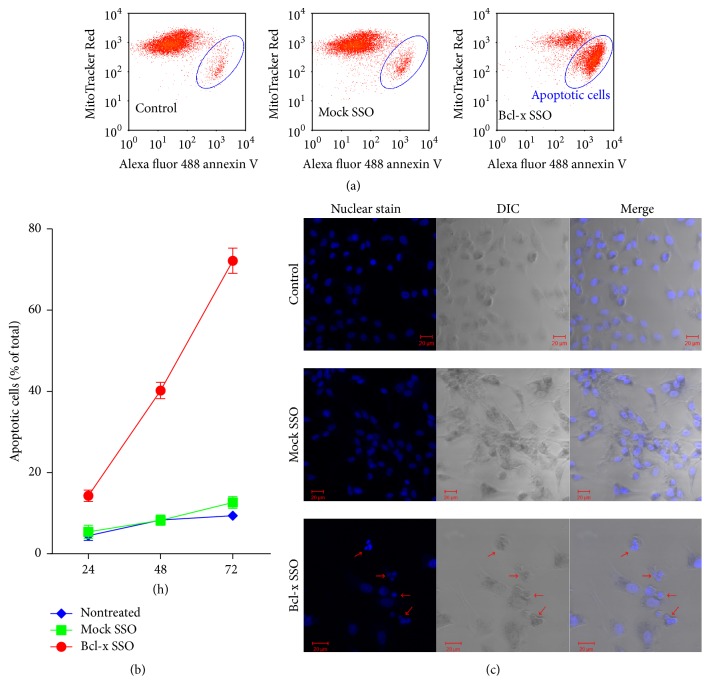
Bcl-x SSO treatment induced apoptosis in HSCs. (a) The cells after Amaxa transfection of SSOs (0.5 nmol) for 48 h were subjected to flow cytometry. The apoptosis kit includes a red dye to indicate mitochondrial membrane potential and a FITC-conjugated annexin V. Cells in Q4 area indicate apoptotic cells, show high level of green fluorescence, and decreased red fluorescence. Bcl-x SSO treatment evidently induced apoptosis. (b) Time-course assay of flow cytometry. Bcl-x SSO treatment induced a significant time-dependent apoptosis. (c) Staining for DNA was performed in HSCs 24 h after SSO treatment. The nuclear changes including condensation and fragmentation indicate classical apoptosis.

To determine if the decreased cell viability observed in Figure 4 was caused by HSC apoptosis, flow cytometry was used to detect the mitochondrial membrane potential and the expression of phosphatidylserine in the treated HSCs. After human HSCs were transfected with 0.5 nmol SSO using an Amaxa machine, apoptosis of these cells was determined by counting the relative amount of annexin V-FITC-positive and MitoTracker® Red-negative cells. Apoptotic cells show higher levels of green fluorescence and lower red fluorescence than healthy cells. As shown in Figure 5(a), Bcl-x specific SSO time-dependently induced apoptosis in human HSCs. At 24 h after SSO transfection, the apoptotic cells were 21% of the total cells. The percentage increased to 53% and 75% at 48 h and 72 h, respectively. In addition, we observed the nuclear changes through confocal microscope. As shown in Figure 5(c), one transfection of 0.5 nmol Bcl-x SSO induced unclear condensation and fragmentation in human HSCs, further supporting the apoptosis mechanism by Bcl-x SSO. By contrast, there was no change in cells treated with the negative control SSO654 at the same dose. These results indicated that the death of HSCs by SSO transfection was through Bcl-x SSO-induced apoptosis.

## 4. Discussion

Stimulating apoptosis of HSCs has been considered as a necessary step to achieve liver fibrosis resolution [6, 12, 13]. Thus, elucidation of the mechanisms for HSC apoptosis and identification of the key players in this process can lead to discovery of new antifibrotic targets and to development of novel therapeutic strategies. The Bcl-2 gene family plays the dominant role in regulation of apoptosis of mammalian cells [29]. Bcl-2, the archetypal member of this gene family, has been indicated to play an important role in HSC apoptosis [2]. However, there are other two important antiapoptotic members in this gene family, Bcl-x and Mcl-1, and they play important roles in some cell types and diseases [29]. The significance of individual Bcl-2 family members in apoptosis resistance depends on their relative expression levels and is specific to cell type. For example, it has been indicated that hematopoietic cancers are dependent upon Bcl-2 for survival or resistance to stress, whereas epithelial cancers rely on Bcl-x_L_ for chemoresistance [30, 31]. In this study, we first evaluated the role of Bcl-x in apoptosis of HSCs by quantifying mRNA expression levels of Bcl-2 and Bcl-x in activated HSCs. The results indicated that expression of Bcl-x is over 83-fold greater than Bcl-2, indicating that Bcl-x may play an important role in HSC apoptosis regulation. Alternative splicing of Bcl-x is an important regulation mechanism. Thus, we measured the relative expression levels of the two splice variants, Bcl-x_L_ and Bcl-x_S_, during passaging of primary culture human HSCs, a process mimicking HSC activation [32]. We observed that the ratio of Bcl-x_L_ over Bcl-x_S_ increased from 18 at passage 1 to 65 at passage 7, indicating an increase in antiapoptotic Bcl-x_L_ expression as well as a decrease in proapoptotic Bcl-x_S_ expression. The change of the ratio of these two members with opposite functions in apoptosis is correlated with the progress of antiapoptosis of HSCs, indicating it may contribute to the resistance to apoptosis of HSCs.

Using antisense to switching alternative splicing is a unique strategy to modulate gene expression and function. By eliminating an overexpressed prosurvival splice variant and inducing an apoptotic splice variant simultaneously, a Bcl-x SSO can potentially achieve greater pharmacological effect than classic antisense or siRNA ONs that can only act on a single function [20, 22]. The results of this study supported this notion. Our data indicated that Bcl-x SSO treatment could directly cause cytotoxicity in human HSCs without other proapoptotic stimuli. By contrast, Bcl-2 silencing by RNA interference could not induce prominent apoptosis [14]. Cells became sensitive only when they were treated with Bcl-2 siRNA and other proapoptotic stimuli such as TNF*α* together [14]. Therefore, the dual functional Bcl-x SSO may have advantage over single functional siRNA. As redirection of alternative splicing using SSO has achieved initial clinical success in treating patients with Duchenne muscular dystrophy [23], this study may lead to a novel molecular therapy for liver fibrosis. Further, the effective stimulation of apoptosis by Bcl-x SSO supported that Bcl-x and its alternate splicing play an important role in HSC apoptosis.

The strategy of stimulation of HSC apoptosis is clinically feasible for treating liver fibrosis only if the apoptosis modulation is specific to HSCs, as hepatocyte death leads to impaired liver function and even exacerbates liver fibrosis. Previous studies have indicated that Bcl-x SSO only causes apoptosis in cells with high expression of Bcl-x_L_ while cells with low levels of Bcl-x_L_ resisted the treatment [33]. Thus, we also measured Bcl-x RNA levels in hepatocytes. Its level is lower than activated HSCs (Figure 1(b)), and thus Bcl-x SSO may not cause apoptosis in hepatocytes. This notion may be supported by an animal study using liposomal delivery of Bcl-x SSO [21]. Liposomal formulation is known to accumulate nonspecifically in the liver, largely into the hepatocytes, due to the fenestrated vasculature [34]. However, liposomal delivery of Bcl-x SSO did not cause liver toxicity, supporting the idea that the hepatocytes may tolerate Bcl-x SSO due to low expression of Bcl-x_L_ [21]. To further enhance the specificity of this strategy, Bcl-x SSO can be targeted to HSCs* in vivo* using targeted delivery systems such as M6P modified system [35]. Specific delivery of the therapeutic entity to HSCs can provide a second safeguard against possible side-effects of Bcl-x SSO in the hepatocytes [36, 37].

In conclusion, the results in this study support that modulating Bcl-x gene alternative splicing by antisense oligonucleotides causes HSC apoptosis and thereby may lead to a novel molecule therapy of liver fibrosis.

## Abbreviations

ECM: Extracellular matrix
HSCs: Hepatic stellate cells
SSOs: Splice-switching oligonucleotides.
